# Homœopathy in Animals

**Published:** 1893-11

**Authors:** 


### HOMŒOPATHY IN ANIMALS.

### Dr. Reed's Paper "Homœopathy in Faithless Animals."

(Extract from Proceedings I. H. A.)

### Discussion.

Dr. Randall—I should say that this was a clear case of animal magnetism. Animals do have faith in their master. And then animals very generally get well without medicinal interference. Sometimes it looks as if medicine did something, and yet, how can we be sure about it? I had a horse that went lame. The lameness grew worse until I gave the animal Rhus-tox. in about the third decimal, and he got well. This seemed an effect of the medicine.

Dr. Reed—There was a necessity for a repetition in this case that I reported. Certainly the remedy did the work, the horse is well to-day. I did not expect one powder to cure the horse, Rhus being a short-acting remedy.

Dr. Hoyne—On the contrary, I think that Rhus is a long-acting remedy. The man who has been poisoned with Rhus-tox. often has symptoms which last seven or eight years or even longer. Dr. Allen treated a case some years ago, where the man had been poisoned fourteen or fifteen years before, and every year about the same time, the symptoms returned. Neihard, who made provings of Rhus, says that for seven years afterward he experienced, every summer, a return of Rhus symptoms.

Dr. Reed—That is undoubtedly true, but in the administration of Rhus in a diseased state, it acts briefly. I have never been able to cure a chronic case of rheumatism with a single dose of Rhus.

Dr. H. C. Allen—When you repeated the remedy why did you not change the potency?

Dr. Reed—We were doing well under the one potency and I did not change it on that account.

Dr. H. C. Allen—I want to report two cases. One was what they call azoturia in horses. The case was attended by a veterinary surgeon who lives a few blocks from me. He is a Harvard graduate, an intelligent man, and a first-class diagnostician. The usual course in such cases is to shoot the animal, as it is considered an incurable disease. As this horse was a very valuable one I went to see if I could do anything for it. I looked over the case pretty carefully with him and gave a dose of Con.$^{cm}$, and greatly to the astonishment of the doctor, cured the horse. Since that time he has relieved a number of cases with Conium and other remedies. The second case which I wish to report is a case of pneumonia in a very valuable animal. I was called on one morning by the veterinary surgeon to consult with him over this horse. The animal had been sick some days and had fallen from weakness, and the veterinary thought the horse would die before night. Two or three symptoms called my attention to Phosphorus. I gave one dose Phos.$^{1m}$, about nine A. M. Before noon the horse was better, but by evening grew worse again, and fell in the stall unable to rise. I gave another powder of Phos.$^{cm}$. Next morning the surgeon reported that the horse was so much better that he was standing up eating. These two cures converted this intelligent surgeon to Homœopathy and he became a matriculant of the Hering College.

Dr. F. O. Pease—Not long after purchasing a horse I discovered that when driving at all fast, he would begin to jerk his head in such a way as would make one imagine that mud or sand was being thrown into his eyes. Two weeks after he had another attack while driving him on a street not at all muddy. He stopped, jerked his head, and I thought was a little unsteady on his feet. Such attacks came on every seven to twelve days. I consulted a veterinary who said it was blind staggers, and the best thing I could do was to sell the animal, as they were never cured. I gave Nux-vomica, and after Belladonna without benefit. Finally I consulted Dr. Butler, who wrote to me that he thought Iodine was the remedy, but by that time I had noticed that the attacks were not apt to come on after a hot day, and that there was a wave-like motion traveling from head to tail. I concluded to give him Glonoinum. He had one severe fit the next day, and has never had any since. From that time his coat, which had been dull, brightened up.

Dr. H. P. Holmes—This discussion calls up several questions in my mind. There is a point I want to emphasize. When a physician reports a case successfully treated, it seems to me an ungrateful thing to ask him why he did not do it some other way. Nor is it quite *apropos* to explain it away, and say the remedy had nothing to do with the cure. The subject we are discussing is the cure of disease in animals who can have no faith, and such cures should be fully reported, for they will have a better effect upon the general public, and upon that part of the profession who do not believe in the high potencies than any other kind of cases. I think most of us have noticed that there is, in some of our most intelligent families, a strong tendency toward the faith cure and toward Christian science which I wish they did not have. One family which I had succeeded in getting away from the old school, began to exhibit a strong drift toward Christian science. I accordingly was anxiously awaiting for an opportunity of showing them that Homœopathy was not all faith.

One of a very fine team of horses belonging to them was taken sick. While I was visiting them the husband asked me if I would object to prescribing for their horse.

The symptoms called for Aconite, and in twenty-four hours he was all right, and that was just the argument I was hoping for to prove my point.

Dr. C. H. Day—It seems to me that the dumb animals do have faith in their masters, a great deal more than our most intelligent people.

Dr. Adams—That might lead to a long discussion as to what is faith, but we will not go into that now. The family dog who had had pups was suffering fearfully from irritation of the genital organs. The owner begged that I would do something for it. As a great favor, I went to see the animal and prescribed Kali-carb.$^{cm}$, which relieved the whole trouble in a few hours. Some months after, all of his dogs were taken sick with influenza, and in this case Arsenicum$^{cm}$ cured them. The Kali-carb.

was prescribed on account of the discharge and the relief from pressure.

Dr. A. R. Morgan—I remember some years ago making a cure on an ox. I wanted to borrow a yoke of oxen to do some work, and I went to see this man who owned the oxen, and who, by the way, was a strong homœopath. He said he would be glad to loan me them, but one of them was very sick. I went out to see the animals. I found one of them a splendid-looking animal, but the other was rather Gothic in his architecture—hair sticking out in every direction, and the yard in which he stood showed every evidence of diarrhœa. It had been under the care of a veterinary. I had never treated an ox, but I said if that was a man I would give him Arsenicum. The stools were thin, offensive, and watery, and occurred particularly after eating.

At that time the highest remedy I carried was the 30th. I gave him a dose, and said, laughingly, I guess he is all right now. Two days after, the man came to my house and said that the ox was much better. He wanted more medicine. The ox did recover shortly, and I used him to do my work.

Dr. J. H. Allen—I have had a little experience with chicken cholera. The chickens were dying, one an hour. The most marked symptoms were great thirst and diarrhœa. I put a powder of Arsenicum in the water and saved all of them, except the dead ones.

